# The effectiveness of the psychosocial empowerment program in early adjustment among adult burn survivors

**DOI:** 10.1186/s12912-024-01700-x

**Published:** 2024-01-16

**Authors:** Evon S. Shokre, Shaymaa Elsayed Mossad Mohammed, Heba Mohammed Mahmoud Elhapashy, Nadia Bassuoni Elsharkawy, Osama Mohamed Elsayed Ramadan, Enas Mahrous Abdelaziz

**Affiliations:** 1 Department of Psychiatric Mental Health Nursing, Faculty of Nursing, Fayoum University, Fayoum, Egypt; 2 Department of Medical-Surgical Nursing, Faculty of Nursing, Mansoura University, Mansoura, Egypt; 3 College of Nursing, Jouf University, Sakaka, 72388 Saudi Arabia; 4 Department of Maternal and New-born Health Nursing, Faculty of Nursing, Cairo University, Cairo, 11562 Egypt; 5 Department of Paediatric Nursing, Faculty of Nursing, Cairo University, Cairo, 11562 Egypt; 6 Department of Psychiatric Mental Health Nursing, Faculty of Nursing, Cairo University, Cairo, 11562 Egypt

**Keywords:** Burn survivors, Psychosocial empowerment, Nurse-led intervention, PTSD symptoms, Recovery strategies

## Abstract

**Background:**

Burns constitute a major global health challenge, causing not only physical trauma, but also significant psychosocial and emotional disturbances. The complexity of these injuries requires comprehensive rehabilitation programs that address both the physical and psychosocial aspects of recovery. Despite advances in medical care, there is a lack of standardized, accessible, and sustainable psychosocial interventions for burn survivors, particularly in the transition from hospital to home. This study aimed to develop and evaluate a nurse-led psychosocial empowerment intervention for early adjustment among burn survivors after hospital discharge.

**Methods:**

The study adopted a quasi-experimental framework. A convenient sample of 80 adult burn survivors was randomly divided into an intervention group, receiving the psychosocial empowerment program, and a control group, continuing standard care from November 2022 to May 2023. The effectiveness of the program was evaluated using various tools that measure satisfaction with appearance, coping abilities, and symptoms of post-traumatic stress disorder (PTSD). The intervention focused on enhancing resilience, self-efficacy, and adaptive coping, through targeted skill building in stress management, adaptability to coping, social reintegration, emotion regulation, and problem-solving.

**Results:**

Participants in the intervention group demonstrated significant improvements in body image satisfaction, coping abilities, and symptoms of PTSD compared to the control group.

**Conclusions:**

The psychosocial empowerment program effectively addressed the psychosocial needs of burn survivors and enhanced their early adjustment after hospital discharge. The findings highlight the critical role of psychosocial support in the rehabilitation of burn survivors and underscore the need to integrate such interventions into standard post-discharge care. Future research should focus on the long-term effects of these interventions and their applicability in diverse settings.

## Background

Burn injuries, which represent a significant global health challenge, are the fourth most common trauma worldwide, after traffic accidents, falls, and interpersonal violence [1–4]. These incidents, which result in substantial mortality and long-term disability, present a significant health crisis around the world [5]. The sudden and impactful nature of burn injuries disrupts not only the physical state, but also the social, psychosocial, and emotional realms of human life, often leading to immediate and lasting challenges [6, 7]. It is estimated that around 1% of the population will experience a burn injury at some point in their lives, and countries such as Egypt report particularly high rates of mortality and morbidity, where up to 40% of severe burn cases result in deaths [7, 8]. Despite strides in medical care and the establishment of specialized burn management facilities, the complexity of burn injuries, including their complications, remains a significant hurdle in inpatient recovery [6, 9, 10].

As medical advancements in burn care have improved survival rates, there is a parallel need for robust rehabilitation programs that address the psychosocial aftermath for survivor’s post-discharge [11–13]. Approximately one-third of major burn survivors report substantial psychosocial symptoms after hospital discharge, encompassing a spectrum of issues such as fear, anger, body image disturbances, post-traumatic stress disorder, depression, anxiety, and social isolation [14, 15]. Such psychosocial complications are not uncommon, with prevalence estimates ranging from 28 to 75% among survivors [14, 16]. These injuries not only physically scarred, but also serve as a harbinger of various long-term psychosocial disorders, which require complete support at all stages of burn care [15, 17].

The literature underscores the critical role of body image and coping strategies in the psychosocial rehabilitation of burn survivors [18]. The psychological trauma from burns is not only due to physical injury, but also to changes in appearance, personality, and coping mechanisms, all of which significantly affect the adjustment of survivors after injury [19–22]. Active problem solving and positive reframing, in contrast to avoidant coping strategies, are associated with a better quality of life and fewer depressive symptoms, highlighting the importance of adaptive coping in recovery [23–25].

Visible scarring and disfigurement can cause social stigma and challenges in public interactions, underlining the need for early interventions that combine psychosocial support with medical treatment to improve general well-being [26–28]. Various non-pharmacological interventions, including psychotherapy, self-care education, and social skills training, have shown effectiveness in mitigating pain and psychosocial difficulties among burn patients [27, 29].

The multidisciplinary approach in patient management is crucial and burn care nurses play a crucial role in addressing the biopsychosocial needs of patients [30, 31]. Their continuous contact with patients requires the design and implementation of educational programs that equip patients with essential skills for effective treatment of their condition [32]. Recent advances in technology and treatment methods, coupled with shorter hospital stays, demand the creation of empowerment programs that address psychosocial care alongside physical care [30, 31]. Such programs are expected to be highly effective in promoting patient participation in the management process, improving their coping skills, and facilitating adaptation to changes after burn [33, 34].

Despite promising evidence for existing psychosocial interventions, current approaches have significant limitations, including a lack of standardization, limited accessibility and generalizability for many patients, high resource demands, and questionable sustainability over time [35–37]. In particular, there are empirically tested theory-driven psychosocial empowerment programs for burn survivors transitioning from the hospital to home [38]. Empowerment models go beyond traditional passive supportive therapy to actively equip patients with the motivational, cognitive, and behavioural skills needed to manage persistent challenges [39, 40]. Although patient empowerment aligns with positive psychology and posttraumatic growth principles, empowerment-based psychosocial programming remains unexplored and untapped to date in burn rehabilitation contexts [32, 41–44].

Therefore, the current study seeks to develop and evaluate an innovative psychosocial empowerment intervention tailored to the realities that burn survivors face after discharge. This nurse-led curriculum focuses on fostering inner resilience and self-efficacy through targeted skill building in areas spanning stress management, coping adaptability, social reintegration, emotion regulation, and problem-solving. We hypothesize that this structured empowerment training will support early psychosocial adjustment for burn patients versus standard care. The findings are expected to delineate an optimized biopsychosocial rehabilitation model that combines evidence-based psychosocial empowerment with existing medical therapies, producing a new gold standard for burn aftercare that promotes survivor self-management and quality of life.

### Research hypotheses

1. Burn survivors who receive the psychosocial empowerment program will report significantly higher satisfaction with physical appearance after the intervention compared to patients who receive standard care alone.
2. Burn survivors who receive the psychosocial empowerment program will demonstrate significantly greater improvements in adaptive coping skills post-intervention versus those undergoing routine care.
3. Burn survivors who complete the psychosocial empowerment curriculum will exhibit significantly greater reductions in post-traumatic stress symptoms following the intervention relative to the control group.

## Methods

### Design

In this study, we adopted a quasi-experimental framework that incorporates rigorous evaluation of the impact of a psychosocial empowerment program on burn survivors. The design includes two key stages: pre- and post-intervention assessment. Participants are divided into two different groups: the intervention group, which receives the psychosocial empowerment program, and the control group, which continues with standard care without additional psychosocial empowerment intervention. This separation allows for a comprehensive comparison between the two groups and allows for a detailed understanding of the effectiveness of the program.

### Setting

The study was carried out at the Burn Center of the Mansoura University Hospital, Dakahlia, Egypt. This research was carried out between November 2022 and May 2023 and used the advanced facilities and specialized care offered by the hospital. The environment has been specifically selected for its extensive burn treatment and rehabilitation services, which provide an appropriate and conducive environment to assess the impact of psychosocial empowerment programs on burns.

### Sample

A convenient sample of 80 adult burn survivors admitted to the Burn Centre at the University Hospital of Mansoura, Egypt, were included in the study. The sample size is based on the analysis of the G * Power 3.1.9.2 software to detect 0.5 medium effects at a power of 0.80 and an alpha level of 0.05 in the intervention. This calculation required 40 patients per group. Participants were randomly assigned to the intervention group for psychosocial empowerment (n = 40) or to the standard care control group (n = 40), using computer generated randomization to ensure impartial group allocation.

The sample size of 40 participants per group was determined based on a power analysis, which indicated that this number was sufficient to detect significant effects given the specific parameters and expected effect size of our research. Although this sample size is slightly smaller than the 45 to 50 participants utilized in related studies [45, 46], it aligns with the general scale of previous research in this area. This clarification ensures a more accurate representation of our methodological approach and its alignment with established research standards in the field.

### Eligibility criteria

#### Inclusion criteria

1. Individuals of all genders are eligible.
2. Patients should be between 18 and 60 years of age.
3. The patient must spend at least one week in the hospital after the burn.
4. Burns should result from accidents or burns caused by electricity.
5. The range of the body surface area affected by burns should fall within 10–40%.
6. Burning levels must be categorized as 1, 2, or 3.
7. Patients must not have any disabilities related to burn injuries.
8. Burn patients who have informed consent and are willing to follow research procedures.
9. Patients should be able to communicate effectively.

#### Exclusion criteria

1. Patients with serious burn injuries and complications.
2. Patients with sensory and motor impairment.
3. Patients with mental retardation or diagnosed with psychiatric disorders, such as depression.
4. People with chronic diseases such as diabetes, liver disease, and heart failure.
5. Patients are currently participating in another burn program.

### Data collection tools

1. **Sociodemographic and Burn Characteristics Questionnaire**: This questionnaire was used to collect data on gender, age, occupation, marital status, educational level, and economic status, as well as details on the incident of the burn, such as the burning agent or the heat source, causes of the burn, grade, and percentage of burns.
2. **Satisfaction with the appearance scale**: Developed by Lawrence et al. (1998) [47], this 14-item questionnaire was designed to assess body image satisfaction in burn patients. The questionnaire was divided into two sections. The social behavioural impact of disfigurement contained 6 questions, and the subjective satisfaction scale contained 8 questions. The responses are scored on a scale ranging from 1 (strongly disagree) to 7 (strongly agree), and the questions from 4 to 11 were scored reversely. The range of scores in this questionnaire was (14–84). A higher score indicated a higher dissatisfaction with body image. The internal consistency was estimated as α = 0.87 in patients with burn injuries [14], while the reliability of the current study measured by internal consistency, Cronbach’s Alpha was 0.958.
3. **Coping with Burn Questionnaire**: Developed by Wildal Brand (2001) [48], this questionnaire measures coping strategies among burn survivors. It consists of 33 items divided into six subscales corresponding to different dimensions of coping: avoidance, self-control, emotional support, instrumental action, adjustment, and optimization of problem-solving (Table 1). Items are rated on a scale of 1 to 4 (1 - does not apply/not used, 2 uses somewhat, 3 uses quite a bit, and 4 uses a great deal). The reliability of the current study was measured by internal consistency; Cronbach’s Alpha was 0.855.

**Table 1:** Content of subscales in the coping with burns questionnaire (CBQ)

CBQ subscales	Content of subscales
Avoidance	Cognitive and behavioral efforts to divert attention away from difficulties or reminders of the accident
Self-control	Restrained expression of feelings
Emotional support	Looking for emotional support
Instrumental action	Instrumental support seeker and instrumental ways of problem solving
Revaluation Adjustment	Statements about changing, adjusting, and restricting thoughts about the accident and lifestyle to feel better
Optimism-problem solving	Strategies such as putting effort into making things work, using cognitive strategies in solving problems, and having a positive outlook on the future.

4. **Post-traumatic stress disorder (PTSD) checklist**: This checklist is used to assess the symptoms of posttraumatic stress disorder. This checklist was translated into Arabic [49], the internal consistency among the Iraq participants was high (alpha = 0.85) [50]. The checklist comprises 20 items; it is rated on a scale of 0 (not at all) to 4 (extremely). A total symptom severity score (range 0–80) can be obtained by summing the scores for each of the 20 items. Questionnaire scores have shown strong internal consistency (α = 0.94 to 0.96) [50]. The current internal consistency, Cronbach Alpha, of the questionnaire was 0.831.

A pilot study was conducted to assess the format, clarity, appropriateness, and completion time of the data collection instruments. In the pilot study, 10 burn patients participated and any necessary modifications were made. The patients involved in the pilot study were then excluded from participation in the main study.

### Ethical considerations

Approval for this study was granted by the Institutional Review Board (IRB) of the Mansoura Faculty of Nursing prior to any participant recruitment or data collection (IRB Number: P.0456). In addition, administrative clearance was obtained from the directors of the Mansoura University Hospital Burn Center. Participation in the study was entirely voluntary. During the informed consent process, patients received comprehensive information about study objectives, procedures, associated risks and benefits, confidentiality safeguards, and their unrestricted right to withdraw from the study at any time without experiencing adverse consequences. To protect your privacy, no identifiable information was collected, and all participants were assigned unique code numbers. All data collected were securely stored in encrypted files, accessible exclusively to authorized study personnel, thus maintaining the highest standards of confidentiality.

Research was carried out strictly following the ethical guidelines outlined in the Declaration of Helsinki, which emphasizes the principles of beneficence, justice, respect, and autonomy of participants. Any significant amendments or adverse events would have been promptly reported to the institutional IRB, as described in the established protocol. Throughout this study, the paramount consideration was the well-being, dignity, and rights of all human participants. The unwavering adherence to these ethical regulations and the transparency in the procedures exemplify the commitment to conducting rigorous and ethically sound research.

### Procedure

In accordance with the official approval received from the Ethical Committee of El Mansoura Faculty of Nursing, El Mansoura University, Egypt, the research team, as academic professionals, initiated contact with the research units and provided a comprehensive explanation of the research objectives and the process of completing the questionnaire. Subsequently, interviews were conducted with participants in the hospital, during which the purpose of the study was articulated and informed consent was obtained. A structured interview format was used to facilitate the completion of the study tools, with all participants responding to questions during the pre-test phase. The duration of each interview session ranged from 15 to 20 min. Following the pre-test phase, participants were randomly assigned to the intervention or control group. The intervention group received standard care such as pain killer, IV fluid administration, antibiotics, dressing, sometimes skin grafting according to the severity of the condition, and physiotherapy in some cases, in addition to the psychosocial empowerment program.

### Psychosocial empowerment program phases

#### Assessment phase (two sessions)

This initial phase included the completion of the baseline assessments (T1) by both groups. Participants were required to complete the demographic information questionnaire and burn characteristics, the satisfaction scale with personal appearance, the Burn coping questionnaire, and the posttraumatic stress disorder checklist. The purpose of the study, the role of researchers and the methods of program delivery were thoroughly explained to ensure the comprehension and cooperation of the participants. After completion of all baseline evaluations, participants were randomly assigned to the intervention group (receiving standard care in conjunction with the psychosocial empowerment program) or the control group (receiving only standard care). The program content for each session was provided to participants in the psychosocial empowerment program at the end of the session.

#### Implementation phase (8 sessions)

The intervention group received the Psychosocial Empowerment Program (PEP), consisting of eight sessions, each session lasting a specified duration. Six of these sessions were conducted during patient hospitalization, and three sessions occurred weekly. Each session accommodated a group of five patients, and the last two sessions were scheduled after discharge at follow-up clinics, ensuring continuity of care. Each session began with a review of the assignments from the previous session and the provision of feedback on the application of the learned skills. Videos and PowerPoint presentations, developed and presented by the researchers, were used to facilitate the acquisition of knowledge and the development of practical skills, including breathing exercises, muscle relaxation, relaxation techniques (PMR), biofeedback, guided imagery, and anger management. The educational content of the program was meticulously prepared based on the existing literature and presented to the study participants.

### Sessions of the psychosocial empowerment program

#### Session 1

Introduction to the program, establishment of a welcoming environment, and explanation of the concept and benefits of psychosocial empowerment for burn survivors.

#### Session 2

Discussion on the psychological impact of burns, teaching practical skills such as controlled breathing and progressive muscle relaxation to manage stress.

#### Session 3

Exploration of different coping mechanisms, focusing on distinguishing between adaptive and maladaptive strategies, improving self-control, and positive self-talk techniques.

#### Session 4

Development of critical thinking skills, focusing on problem solving and decision-making strategies, tailored to assist participants in daily life challenges.

#### Session 5

Guidance on social adjustment, including techniques for effective communication, relationship building, and strategies for smooth reintegration into the community.

#### Session 6

Pre-discharge session that emphasizes self-care, with an introduction to non-pharmacological pain management techniques, aimed at empowering participants in their recovery journey.

#### Session 7

Addressing the management of negative emotions such as anger and despair, providing practical methods for emotional regulation and control.

#### Session 8

Fostering a positive mindset, focusing on building optimism and resilience, essential for long-term psychosocial well-being.

Homework assignments were an integral part of each session to ensure the application of the skills acquired from previous sessions. Before discharge from patients, researchers confirmed the date and time of follow-up meetings in outpatient clinics. Additionally, reminders were sent one day before each session to ensure patient attendance.

#### Evaluation phase (two sessions)

Post-test evaluation questionnaires (T2) were administered immediately after the intervention to all participants, using the same study tool questionnaires. These post-test scores were compared with the pre-test scores to assess the effectiveness of the psychosocial empowerment program in helping early adjustment among burn patients.

A concise handbook containing all the information and skills taught during the program sessions was provided to participants in both groups after the program was completed.

### Statistical analysis

All data were analysed using IBM SPSS Statistics software version 23.0. Descriptive statistics, including means, standard deviations, and frequency distributions, were examined for study variables and demographic characteristics. The Kolmogorov-Smirnov test verified that the distribution of continuous variables met the normality assumptions before proceeding with parametric analyses. Differences in sociodemographic and burn characteristics between the intervention and control groups were compared using Chi-square tests for categorical variables and independent sample t tests for continuous variables. Paired sample t-tests were performed to determine within-group changes in study outcomes from baseline to post-intervention. Monte Carlo method used to provide accurate *p*-values even when the expected frequencies in the contingency tables are small, which is often the case in medical research with limited sample sizes.Effect sizes were calculated using Cohen’s d to evaluate the magnitude of group differences and changes over time. Multiple linear regression analysis identified the strongest predictors of psychosocial adjustment outcomes while controlling for potential confounders. Statistical significance was determined at *p* < 0.05 across all analyses.

## Results

### Demographics and burn characteristics of the control and intervention groups

Table 2 summarizes the demographic characteristics of 80 burn patients divided equally into control and intervention groups. Both groups had similar mean ages, with no significant age or gender differences. However, notable distinctions were found in education and marital status. The intervention group had a higher proportion of participants with secondary education (50%) compared to the control group (25%), and fewer married individuals (one-third in the intervention group vs. two thirds in the control group). The occupational status was similar between the two groups. Fire was the main cause of burns in both groups, and there were no significant differences in the degree or percentage of burns. This table provides essential insights into the demographic composition of the study cohort, highlighting the significance of education and marital status in the research context.

**Table 2:** Demographics and burn characteristics of the control and intervention groups

Frequency variables	Control group (n = 40)		intervention group (n = 40)		χ^2^	***p*** value
	No	%	No	%		
**Age**						
Mean ± SD	33.88 ± 10.65		32.45 ± 10.11		t = 0.614	0.541
**Sex**						
Male	23	57.5%	18	45.0%	1.251	0.263
Female	17	42.5%	22	55.0%		
**Education**						
Preparatory and earlier	21	52.5%	9	22.5%	8.333^*^	0.016*
Secondary education	10	25.0%	20	50.0%		
University	9	22.5%	11	27.5%		
**Marital status**						
Single	14	35.0%	24	60.0%	6.087^*^	^MC^p= 0.034*
Married	25	62.5%	14	35.0%		
Other: separated or widowed	1	2.5%	2	5.0%		
**Job**						
working	20	50.0%	16	40.0%	2.717	0.257
not working	12	30.0%	19	47.5%		
student	8	20.0%	5	12.5%		
**Cause of burn**						
Fire	24	60.0%	15	37.5%	5.834	^MC^p= 0.097
Water or boiling liquid	10	25.0%	12	30.0%		
Electricity	6	15.0%	10	25.0%		
Chemical	0	0.0%	3	7.5%		
**Degree of burn**						
First	7	17.5%	14	35.0%	4.091	0.129
Second	23	57.5%	21	52.5%		
Third	10	25.0%	5	12.5%		
**Percentages of burn**						
10–20%	28	70.0%	26	65.0%	0.228	0.633
20-40%	12	30.0%	14	35.0%		

**χ**^**2**^: Chi-square test, **MC**: Monte Carlo

### Comparison of pre- and post-intervention outcome measures in control and intervention groups

Table 3 provides a detailed comparison of outcome measures in the control and intervention groups before and after the intervention. The levels of satisfaction of the control group (satisfaction) showed minimal change before and after the intervention, with no statistically significant difference (t1(*p*) = 0.333, *p* = 0.741). In contrast, the intervention group showed a substantial increase in satisfaction after the intervention (t1 (*p*) = 8.626, *p* < 0.001), with a notable large effect size (Cohen’s d = 2.763). Similarly, for general coping (Overall coping), the control group showed marginal changes before and after the intervention, which were not statistically significant (t1(*p*) = 0.973, *p* = 0.337). On the contrary, the intervention group showed a significant improvement in general coping after the intervention (t1(*p*) = 4.090, *p* < 0.001), accompanied by a moderate effect size (Cohen’s d = 1.310). Regarding the scores for post-traumatic stress disorder (PTSD), the control group showed minimal variation before and after the intervention (t1(*p*) = 0.561, *p* = 0.578). On the contrary, the intervention group experienced a significant reduction in PTSD scores post-intervention (t1(*p*) = 2.695, *p* = 0.010), with a moderate effect size (Cohen’s d = 0.863). These findings highlight the substantial impact of the intervention on the intervention group, particularly in terms of increased satisfaction, improved coping, and reduced PTSD scores.

**Table 3:** Comparison of pre- and post-intervention outcome measures in control and intervention groups

Group	Before intervention (n = 40)	After intervention (n = 40)	t_1_	***P***	Effect size (Cohen’s)
	Mean ± SD	Mean ± SD			
**Satisfaction**					
**- Control**	78.10 ± 23.48	76.48 ± 2.09	0.333	0.741	0.107
**- Study**	80.27 ± 21.9	53.55 ± 12.28	8.626*	< 0.001*	2.763
**t**_**2**_ **(p)**	0.428 (0.670)	5.736*(< 0.001*)			
**Overall coping**					
**- Control**	89.93 ± 20.68	91.0 ± 21.20	0.973	0.337	0.312
**- Study**	85.18 ± 19.07	99.48 ± 10.21	4.090^*^	< 0.001^*^	1.310
**t**_**2**_ **(p)**	1.068(0.289)	2.278^*^ (0.027^*^)			
**Post-traumatic stress disorders scores**					
**- Control**	60.05 ± 23.53	59.23 ± 22.93	0.561	0.578	0.180
**- Study**	57.53 ± 14.64	50.20 ± 12.13	2.695^*^	0.010^*^	0.863
**t**_**2**_ **(p)**	0.576(0.566)	2.200^*^(0.032^*^)			

t_1_Paired t-test comparing between before and after-intervention in each group

t_2_Student t-test for comparing between control and study in each before and after intervention

*: Statistically significant at *p* < 0.05

### Factors predicting burn patient coping, satisfaction, and PTSD scores in multiple regression models

Table 4 offers a compelling statistical insight into the variables that influence psychological outcomes in burn patients. It elucidates how demographic and burn-specific factors, including age, sex, marital status, burn severity, and extent of burn, uniquely contribute to coping mechanisms, satisfaction levels, and PTSD scores. In particular, significant predictors such as sex (female) and marital status (married) demonstrate strong associations with these outcomes. The high R² value indicates a substantial explanatory power of these factors over the variance in psychological outcomes. This comprehensive analysis, underscored by robust statistical measures, is instrumental for healthcare professionals in understanding and addressing the complex psychosocial needs of burn patients, thus facilitating more effective and personalized rehabilitation strategies.

**Table 4:** Factors predicting burn patient coping, satisfaction, and PTSD scores in multiple regression models

Variable	Coefficient B	SE	β	t	***p***	R²	F	***p*** (Model)
Age	0.352	0.129	0.183	2.717	0.008	0.693	33.447	< 0.001
Sex (Female)	19.697	3.091	0.498	6.372	< 0.001	0.693	33.447	< 0.001
Marital status (Married)	9.193	2.700	0.264	3.405	0.001	0.693	33.447	< 0.001
Degree of burn (Third)	7.728	2.394	0.247	3.228	0.002	0.693	33.447	< 0.001
Percentages of burn	-2.170	2.811	-0.051	-0.772	0.442	0.693	33.447	< 0.001

## Discussion

The key objective of this quasi-experimental study was to develop and evaluate a nurse-led psychosocial empowerment intervention to increase adjustment among burn survivors after hospital discharge. The curriculum focused on fostering resilience, self-efficacy, adaptive coping, and social reintegration through structured skill development. As hypothesized, burn patients who received empowerment training demonstrated significant improvements in all psychosocial outcomes, including satisfaction with body image, coping skills, and symptoms of PTSD compared to the control group.

Satisfaction with appearance among burn survivors was the most prominent predictor of psychosocial functioning, related to challenges with mental and physical health. Previous studies have noted the importance of body image satisfaction in the development and maintenance of psychosocial adjustment long after burn injury [14, 17, 51]. This also agrees with [52], who also suggested that strategies to address appearance satisfaction are vital for patient rehabilitation. From our results, the empowerment program improves the patient’s satisfaction with the appearance. A possible explanation of these findings is that our rehabilitation program considers body image disturbance a priori and helps the patient practice self-talk strategies to accept changes and deal with them in highly confidential ways. In addition, we help the patient to get in touch with many civil associations concerned with the rehabilitation of burn patients and provide them with various methods of support.

Our results corroborate the findings of a previous study that uses the Delphi method to empower the patient and report improving overall patient satisfaction, including general appearance [53]. There are similarities between our results and those of [54] who found a significant difference between the mean scores of body image before and after educational interventions. This result also agrees with [55] who reported that burn survivors of the intervention group have higher levels of confidence and acceptance. Another study established that there are statistically significant differences between the study and control groups after intervention in ‘all items of psychosocial health’, including satisfaction with appearance [56]. As mentioned in the recommendation of [57], the healthcare policymakers must adopt some strategies to improve resilience and self-efficacy in burned patients to enable them to cope effectively with the stressful conditions they face as a result of their injuries.

Another important finding regarding is that there is an induces significant changes regarding study participant over all coping. We attribute this result to the fact that the program included many axes, through rehabilitation with exercises, care for the burn site, using relaxation exercises, and writing a plan to manage challenges and difficulties. All of this had a positive impact on the general adjustment of the patient. This finding was consistent with [58] who used the multimedia intervention to empower burn patients and improve their coping. There are similarities between our findings and those of [59]that represent a significant difference between groups and scores in intervention group on coping and anxiety. Another study confirms that the rehabilitation intervention was associated with the psychosocial function of the patients and was significantly improved over the control group [60]. However, the findings of the current study differ from [61] who reporting that there were no significant differences in measured coping self-efficacy after protocol implementation.

Survivors of severe burns are more likely to experience post-traumatic stress disorder. Previous studies have noted the importance of psychosocial intervention programs to improve positive coping strategies and decrease PTSD [62]. In our program, the lowest effect was a moderate change in mean scores of post-traumatic stress disorders. It seems possible that our results are due to memories of traumatic events or images that appear continuously in patient dreams, resulting in increased stress symptoms. According to previous similar studies [63] who investigate the effect of web-based training on the level of post-traumatic stress symptoms and reporting that the mean score of total traumatic stress and its subscales decreased in the experimental group and increased in the control group and the difference was statistically significant and the results of [64] showed that the treatment program increased the re-evaluation component (as a positive emotion regulation strategy) and decreased repression scores (as a negative strategy) in PTSD patients due to burn injuries. A strong relationship between PTSD and intervention levels before and after intervention has been reported in the study conducted by [65].

Another finding that stands out from the results reported earlier is that there were significant effects of effective coping on improving participants’ levels of satisfaction and on decreasing scores for post-traumatic stress disorders; justification for this result that our participants gained positive power from the empowerment program and coped to complete responsibilities for their life and family, as for the constant PTSD with patients, the result of fear of confronting others and fear of being exposed to the same circumstances again. This finding was also reported by [66], who found that there were statistically significant negative correlations of the burn patient coping strategies score with post-traumatic stress disorder. These results reflect those of [67] who also found that the protocol group reported higher self-efficacy scores for coping and decreased anxiety. These differences can be explained in part by the intervention of [68] who reported successful improvement of outcomes, especially attitudes toward appearance of PTSD. Also [69] report the same results. In examining the impact of various factors on coping mechanisms among burn patients, this study underscores the intricate relationship between patient characteristics and their coping strategies. Findings showing that gender, marital status, burn severity, and age significantly influence coping, resonate with, and extend the existing literature. Specifically, we draw parallels with studies that emphasize the role of burn severity, affected body surface, and burn location in psychiatric outcomes [70–73]. This alignment with existing research underscores the multifaceted nature of coping in burn patients.

In contrast, results diverge from studies that position age as the sole determinant of coping, or those that undermine the importance of degree of burn [74–77]. This discrepancy invites a deeper exploration of coping dynamics, suggesting a more complex interplay of factors than previously understood. For example, the increased anxiety about social appearance observed in single patients, compared to their married counterparts, further highlights the role of social factors in coping. Therefore, this study contributes to a nuanced understanding of coping mechanisms, challenges, and refinements of existing paradigms in burn care research.

Abazari et al. [78] reported that the severity of burns, the total body surface area involved, the site of burns, and the depth of the burns all play a role in the development of psychiatric problems. In the same line with the results of [79], who reported that the increase in burned skin surface directly affects overall health. Similarly, the results of [57] reported that clinical characteristics such as the total percentage of burns, the degree of burns and the individual characteristics of patients affect the coping.

The findings of the current study contradict those of [57] who suggested that the age of the patient was the only influencing factor for coping. Similarly, the results are contrary to [57] who found that the degree of burn was not a significant factor in coping. Conversely, [57] discovered that levels of social appearance anxiety were higher among single participants compared to married individuals in their study. Furthermore, [57] reported a significant positive correlation between the demographic variables of education and financial status among burn patients.

## Conclusions

The quasi-experimental study successfully developed and evaluated a nurse-led psychosocial empowerment program for burn survivors’ post-hospital discharge. The intervention effectively enhanced resilience, self-efficacy, and adaptive coping. Significant improvements in body image satisfaction, coping abilities, and PTSD symptoms were observed in burn patients who received empowerment training, compared to the control group. The findings of the study underscore the critical role of psychosocial support in rehabilitation and the importance of addressing body image satisfaction and coping strategies in burn survivors.

### Practical implications and future directions

The practical implications of this study are significant for clinical practice, particularly in the rehabilitation of burn survivors. The success of the nurse-led psychosocial empowerment program suggests that such interventions should be integrated into standard post-discharge care to improve patient outcomes. To facilitate implementation, nursing administrators and managers will need to prioritize staff training in evidence-based psychosocial counseling, make workload adjustments that provide dedicated time for emotional support, and revise policies to formally incorporate psychosocial assessments and follow-up care. Strengthening partnerships with mental health providers and pursuing alternative funding streams may also help obtain resources to sustain long-term programs. At the health policy level, the findings highlight the urgent need to recognize comprehensive psychosocial rehabilitation as an essential component of burn recovery. The development of national clinical guidelines and accreditation standards mandating holistic biopsychosocial care is imperative to drive greater system change.

In terms of future research, it is essential to continue exploring the long-term impacts of empowerment interventions in diverse settings and populations. Multi-site effectiveness trials and implementation studies will clarify adaptability and scalability issues to inform wider uptake globally. Ultimately, advancing this research agenda has the potential to establish nurse-led psychosocial empowerment as the gold standard to support burn survivors during the challenging transition from hospital to home.

### Limitation

1. The study was carried out with a specific sample in a single geographical location, which could limit the applicability of the results to other populations.
2. The reliance on self-reported measures may introduce response bias.
3. The study focused primarily on immediate outcomes; long-term effects of the intervention remain unclear.
4. Without long-term follow-up, it is difficult to assess the sustainability of the benefits of the intervention.

## Data Availability

The datasets used and/or analysed during the current study are available from the corresponding author on reasonable request.
